# A Comprehensive Investigation on Catalytic Behavior of Anaerobic Jar Gassing Systems and Design of an Enhanced Cultivation System

**DOI:** 10.3390/bioengineering11111068

**Published:** 2024-10-25

**Authors:** Fatih S. Sayin, Hasan Erdal, Nurver T. Ulger, Mehmet B. Aksu, Mehmet M. Guncu

**Affiliations:** 1 Electrical–Electronics Engineering, Faculty of Technology, Marmara University, 34854 Istanbul, Turkey; herdal@marmara.edu.tr; 2 Medical Microbiology, School of Medicine, Marmara University, 34854 Istanbul, Turkey; nulger@marmara.edu.tr (N.T.U.); baksu@marmara.edu.tr (M.B.A.); 3 Institute of Health Sciences, Marmara University, 34865 Istanbul, Turkey; mmguncu@gmail.com

**Keywords:** cultivation of anaerobes, McIntosh and Fildes method, heterogeneous catalysis, microkinetic modelling, extremum seeking

## Abstract

The rapid and reliable diagnosis of anaerobic bacteria constitutes one of the key procedures in clinical microbiology. Automatic jar gassing systems are commonly used laboratory instruments for this purpose. The most critical factors affecting the cultivation performance of these systems are the level of residual oxygen remaining in the anaerobic jar and the reaction rate determined by the Pd/Al_2_O_3_ catalyst. The main objective of the presented study is to design and manufacture an enhanced jar gassing system equipped with an extremum seeking-based estimation algorithm that combines real-time data and a reaction model of the Pd/Al_2_O_3_ catalyst. The microkinetic behavior of the palladium catalyst was modeled through a learning-from-experiment methodology. The majority of microkinetic model parameters were derived from material characterization analysis. A comparative validation test of the designed cultivation system was conducted using conventional gas pouches via six different bacterial strains. The results demonstrated high cell viability, with colony counts ranging from 1.26 × 10^5^ to 2.17 × 10^5^ CFU mL^−1^. The favorable catalyst facets for water formation on Pd surfaces and the crystal structure of Pd/Al_2_O_3_ pellets were identified by X-Ray diffraction analysis (XRD). The doping ratio of the noble metal (Pd) and the support material (Al_2_O_3_) was validated via energy-dispersive spectroscopy (EDS) measurements as 0.68% and 99.32%, respectively. The porous structure of the catalyst was also analyzed by scanning electron microscopy (SEM). During the reference clinical trial, the estimation algorithm was terminated after 878 iterations, having reached its predetermined termination value. The measured and modelled reaction rates were found to converge with a root-mean-squared error (RMSE) of less than 10^−4^, and the Arrhenius parameters of ongoing catalytic reaction were obtained. Additionally, our research offers a comprehensive analysis of anaerobic jar gassing systems from an engineering perspective, providing novel insights that are absent from the existing literature.

## 1. Introduction

Automated jar gassing systems are essential components of anaerobic cultivation processes, linking clinical microbiology and medical engineering. Their applications extend to diverse fields, including pathology, food engineering, protein research, etc. Furthermore, these systems provide a reliable tool for creating an anoxic environment for isolated bacterial specimens that are being clinically evaluated [1,2].

The famous pepper tube experiment of Antony van Leeuwenhoek represents the earliest documented evidence of anaerobic life in the scientific literature [3]. Following Leeuwenhoek’s discovery, the first taxonomic research based on the oxygen tolerance of the microorganisms was carried out by Louis Pasteur in 1863, and Pasteur also introduced the terms aerobes and anaerobes for the first time [4]. From these pioneering studies to the present day, exploration and identification of new members of anaerobes continue.

All microorganisms, including bacterial species, need to produce some kind of fuel to sustain their vital functions. In accordance with metabolic requirements, bacterial cells utilize a variety of mechanisms for these purposes, including aerobic respiration, anaerobic respiration and fermentation. At the processing stage of nutrients, aerobes use a series of oxidation reduction reactions to create energy. During the reduction of metabolic oxygen, some kinds of reactive oxygen species, such as superoxide and hydrogen peroxide, can be produced, and these end products impose oxidative stress on bacteria, which leads to various imbalances at the cellular level. Aerobes are naturally resistant to oxidative stress and are able to handle high-level toxic intermediates. In contrast, anaerobes do not have any defense mechanism against oxidative stress sources, which is the most significant factor underlying the oxygen intolerance of anaerobic microorganisms. Because of those reasons, anaerobes have developed a fermentative metabolism. Furthermore, the metabolic end products of fermentation reactions, such as hydrogen, carbon dioxide, acids, and alcohols, are essential components for all living species. From this perspective, anaerobes can be regarded as microscale production facilities, playing a significant role in many recycling processes in nature [5].

Anaerobic bacteria require an oxygen-free atmosphere to survive; however, the microbial community of anaerobes includes numerous species that are sensitive to oxygen in varying degrees. According to their tolerance levels, these microorganisms are classified as obligate, aerotolerant, and facultative in general [6,7,8]. As indigenous members of bacterial microflora, anaerobes are found in inhabited regions of human bodies that include the oral cavity, nasal airways, trachea, pulmonary tracts, and outer portion of the genital areas [9]. Low-oxygenat tissue regions such as the intestinal and genitourinary tissues contain a considerable amount of anaerobic bacteria, as is expected. The presence of anaerobes in the openings of the body, such as the nose, mouth, or larynx, is fundamentally related to the oxygen-consuming metabolic activities of aerobes and facultative bacteria. These microflora states can be seen as environments established on mutual beneficiality [10].

In general, anaerobic bacteria are useful members of the bacterial flora. On the contrary, under exceptional circumstances (e.g., surgical interventions, breakdown of the mucosal barriers, penetration of normally sterile tissue sections by various injuries), anaerobic bacteria can pose a significant risk to human hosts. When they gain access to unusual microhabitats, anaerobic bacteria can colonize these areas rapidly and cause severe infections [11]. Due to their accompanying characteristics, anaerobic bacteria often participate in polymicrobial infections. This situation represents one of the most challenging issues in anaerobic bacteriology and affects the isolation processes directly. During the pathological examinations of anaerobes, clinicians must be armed with a high level of implementation knowledge and great attention. The key principle for distinguishing anaerobes within a mixed infection is to maintain a high degree of suspicion from the beginning of the diagnosis to the end of the treatment [12].

The necessity of personal effort is not the only compelling factor in the isolation, cultivation, and diagnosis of anaerobes. Even today, in this era of advanced technology, only a minority of clinical microbiology laboratories have sufficient equipment to identify anaerobes and perform periodic control procedures [13]. The circumstances just mentioned dictate a conscious ignorance of anaerobes in the clinical fields, as can be clearly seen. These kinds of dissociative approaches also reduce the effectiveness of treatments while increasing the antibiotic resistance of pathogenic anaerobes over time [14]. However, the limited availability of resources and the associated costs can be considered as primary reasons for the insufficient examination of anaerobes in clinical institutions. Additionally, most research related to anaerobic bacteriology equipment is conducted within the medical community. The number of multidisciplinary studies focusing on evaluating and improving the performance of this equipment is quite limited [15,16].

The primary objective of our study is to examine the clinical performance of anaerobic cultivation equipment from an engineering perspective and to enhance their capabilities. In order to achieve this goal, a novel anaerobic jar gassing system that combines real-time vacuum measurements with a microkinetic reaction model to predict changes in the jar atmosphere was developed. An extremum seeking-based iterative learning algorithm was constructed to identify the rate parameters of catalytic reaction and embedded to control unit software. The Pd-doped anaerobic purification catalyst model was built in the context of statistical mechanics and computational chemistry based on material science analysis and used to obtain initial parameters of the estimation algorithm. The jar gassing system is regarded as a catalytic reactor, and the mathematical expression of the concentration values changing due to the gas exchange cycles is presented for the first time in the literature. In order to achieve greater consistency between the model outputs and the actual catalytic outputs, the atmospheric gas composition and the anaerobic gas mixture were analyzed via gas chromatography, and the actual percentages of gases used for the calculations were determined.

Our system design was generally focused on the growth of clinical pathogenic microorganisms that can be easily proliferated on solid media and isolated via standard clinical microbiological procedures. Moreover, the selected organisms are characterized by well-documented pathogenic properties. The main motivation for the selection of this framework is the selective nature of the solid medium, which allows for effective isolation and visual assessment of different types of bacterial colonies causing mixed infections, which are frequently encountered during the pathogenic evaluation of anaerobes [17,18]. Nevertheless, certain anaerobic microorganisms, such as methanogens, can only be cultivated effectively in liquid media. The methodology behind the preparation of these types of mediums can be attributed to the well-known study of the Robert Hungate [19]. The importance of the specialized equipment that is used to create anoxic environments like our system cannot be overestimated for all the mentioned media, including the Hungate’s roll tube. The anaerobic archaea are another group of organisms that require a specific gas atmosphere for growth [20]. The isolation of anaerobic archaea from clinical samples is frequently challenging, and they are not included in standard bacteriological tests. These microorganisms are frequently found in the human microbiota and constitute an important part of the probiotic population [21,22]. In studies examining the pathogenic potential of archaea, it has been observed that although they are unable to cause infection independently, they exhibit high-density growth in areas where complications arise and contribute to methane formation [23]. The role of archaea as indirect contributors to various diseases remains a subject of intensive research [24,25]. Besides these clinical properties, anaerobic archaea are playing an increasingly important role in gut microbiome-related research, particularly in the development of novel antibiotics and biotherapeutics [26]. Probiotics derived from archaea represent a promising alternative to synthetic antibiotics, which have been associated with an increase in antimicrobial resistance on a global scale [27].

The present status of the jar gassing system can be described as intermediate between the experimental prototype and the commercial equipment. Moreover, the system can be evaluated for different types of cultivation equipment. By incorporating additional apparatuses, the system can be adapted for diverse applications. As an example, a stand can be added to allow Hungate tubes to be placed in the jar. Additionally, a new atmosphere generation option can be included for capnophilic bacteria by revising the pneumatic subsystem. The archaea or methanogenic cultivation mode can be integrated into the device. These options were regarded as future investigations by our research team.

The following sections constitute the overall framework of the presented study. Section 2 presents a comprehensive overview of the commonly used methods for the cultivation of anaerobes; Section 3 includes a list of the materials used and the methods presented; Section 4 highlights the results; and Section 5 concludes the principal findings.

## 2. Cultivation of Anaerobes

In clinical practice, all bacterial species that have the potential to cause disease or infection, regardless of which class they belong to, are subjected to examination and evaluation in laboratory areas where appropriate physical (light intensity, wavelength, temperature, humidity, etc.) and chemical (gas atmosphere, medium, etc.) conditions are provided. Pathology samples taken from individuals struggling with a bacterial infection are processed within the framework of a specific application procedure for the isolation of species that cause complications. Therefore, the most appropriate treatment methods, drug types, and doses to facilitate a positive progression of the individual’s current clinical situation are identified. This approach optimizes the efficacy of treatment while minimizing the risk of side effects [2].

**Figure 1:**
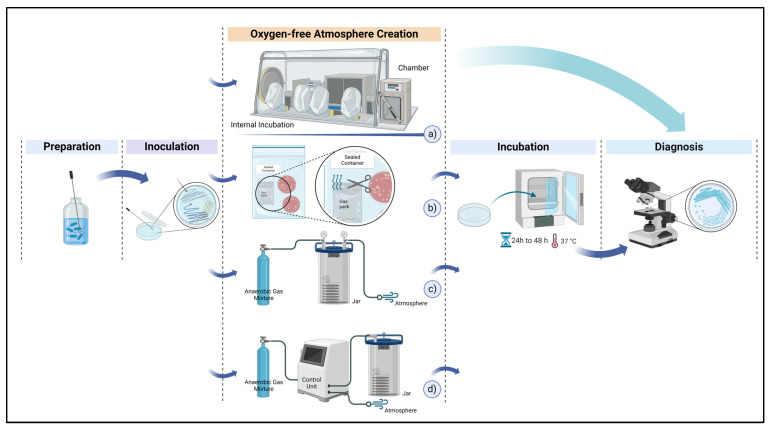
Overview of anaerobic cultivation procedures and atmosphere creation methods: (**a**) anaerobic chamber, (**b**) anaerobic bag, (**c**) anaerobic jar, (**d**) evacuation–replacement device.

Cultivation is the most commonly used method in clinical microbiology. This process involves the preparation of samples collected for clinical evaluation, inoculation into media suitable for different bacterial species, incubation at body temperature, and examination of the growing colonies in terms of both quality and quantity [28]. An illustrative representation of cultivation steps and methods is given in Figure 1.

The most important physical conditions that influence the growth of bacterial cultures following their inoculation into the medium are ambient temperature and the gas atmosphere. Similar to all living organisms, processes such as energy production, protein synthesis, maintenance of pH levels, and so forth, which are essential for bacteria to sustain their vital activities, depend on chemical reactions occurring within the microorganism. In order for these reactions to produce the correct outputs and for the enzymes to function optimally, it is essential to maintain the incubation environment at precisely body temperature [29].

Oxygen, hydrogen, nitrogen, and carbon dioxide gases are among the essential metabolic inputs processed by chemical reactions, as previously mentioned. Therefore, providing the gas atmosphere that the bacterial specimen to be cultured requires for proliferation is one of the crucial stages of cultivation. During the incubation of anaerobic, microaerophilic, and capnophilic bacterial species, it is necessary to utilize specialized equipment to regulate the gas atmosphere of the incubation site in accordance with the requirements of the bacteria [30]. The conventional cultivation methods used in anaerobic bacteriology are outlined in Table 1.

### McIntosh and Fildes (Evacuation–Replacement) Method

The evacuation and replacement method, which is commonly referred to as the McIntosh and Fildes anaerobic jar method, is regarded as a landmark study in anaerobic bacteriology [33]. Since the initial publication of Paul Fildes and James McIntosh’s research in 1916, the evacuation and replacement methods have become a widely used system for creating anaerobic atmospheres [34,35]. The methodology proposed by McIntosh and Fildes enabled more reliable cultivation and also introduced sophisticated apparatuses suitable for advanced and systematic research. In comparison to the techniques from its period, such as the candle jar method, this invention represents a significant advancement in the cultivation of anaerobes.

Fundamentally, the evacuation and replacement method is based on manual gas exchange procedures. During the execution of these procedures, initially, the oxygen-rich atmospheric gas mixture within an airtight jar is ejected to a certain degree of vacuum by a manually operated vacuum pump. Then, the jar is filled with an anaerobic gas mixture (85% N_2_, 10% CO_2_, 5% H_2_) until it reaches atmospheric pressure, utilizing a gas tank. These sequences are repeated a specific number of times. Following the completion of the gas exchange cycles, the oxygen gas concentration within the jar is reduced to below 0.2 percent. This residual oxygen is bonded to hydrogen that comes from the anaerobic gas mixture and transformed into water with the assistance of a palladium catalyst, which is placed within the jar. Consequently, while the jar atmosphere reaches anaerobic conditions, the moisture requirement of the bacteria is satisfied. During all evacuation and replacement cycles, the level of vacuum pressure never decreases to the absolute vacuum value, because the vacuum tension that is posed to the cell membrane of the bacteria can be very destructive, and that is also the main idea behind the cyclic gas exchange.

**Figure 2:**
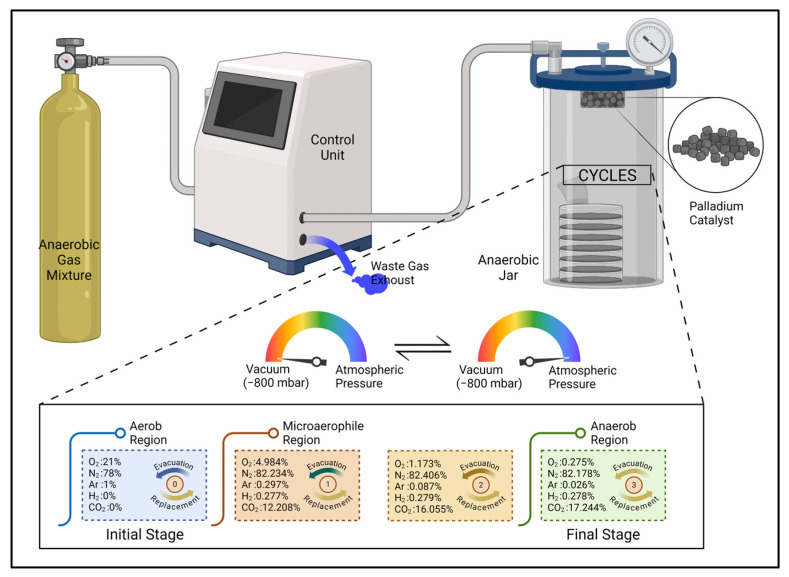
Automatic jar gassing system.

In the original version of the McIntosh and Fildes method, a lab technician or clinician uses several manually operated valves and a pressure manometer for visual feedback to manage the gas exchange processes. These control components are assembled into anaerobic jar lids. The jars used for anaerobic cultivation are frequently manufactured from polycarbonate, which permits visual inspection. Today, a number of conventional jar gassing systems have been developed that are based on the McIntosh and Fildes method and are designed to provide an automatic gas exchange. Such systems typically include a control unit with a user interface, and all evacuation and replacement operations are coordinated by electro-pneumatic components. Furthermore, the use of automated jar gassing systems reduces the necessity for trained clinical personnel and minimizes the effect of man-made errors. The main components of a jar gassing system and a detailed description of cyclic gas exchange are depicted in Figure 2.

Beyond the overall electromechanical structure of these systems, the palladium catalyst is a key element that must be emphasized. The catalytic reaction accelerated by the palladium catalyst is essentially an oxygen and hydrogen recombination reaction. The overall reaction equation is given in Equation (1) [36,37].

(1) $$H_{2}\left(g\right)+\frac{1}{2}O_{2}\left(g\right)\to \;H_{2}O,\;{\;\Delta H}_{R\;}=-244.5\frac{kJ\;}{mol}$$

Such catalysts are typically described in the chemical literature as heterogeneous catalysts. The surface reactions that occur between the gas species (e.g., O_2_ and H_2_) present in the jar atmosphere are generated over the porous structure of the catalysts, thereby providing a large-scale solid–gas interface. Transition metals palladium and platinum are frequently used to manufacture heterogeneous catalysts. The heterogeneous catalysts used for the creation of oxygen-free atmospheres contain approximately 0.5 percent palladium. Additionally, aluminum oxide constitutes the major portion of the catalyst body as a support material. These pellet-shaped catalyst particles are usually packaged in a steel sachet and used as accessories. After a certain number of uses, palladium catalysts reach saturation, which reduces their catalytic activity. To discharge the catalyst surfaces, the sachets are baked at a temperature of approximately 180 °C.

## 3. Materials and Methods

This section emphasizes all the design, production, and validation phases of our evacuation and replacement system and estimation software. Details of these studies are presented on three main topics.

### 3.1. Automatic Jar Gassing System Design and Fabrication

The primary objective of this study is to enhance the performance of evacuation and replacement-based cultivation systems. To achieve this goal, we designed an experimental cultivation system that allows us to conduct a comprehensive research project encompassing both the clinical applications and the engineering perspective on these systems. Furthermore, the system enables us to control and observe all system components.

#### 3.1.1. Hardware and Software Design

The interval between the initial design study and the production of the cultivation device’s final version is approximately four years. During this period, the control unit, anaerobic jars, and other accessories were revised based on the results of clinical experiments and feedback from practitioners. Before integrating advanced features into our system, we attempted to achieve the capabilities of conventional systems. The current cultivation systems frequently used in anaerobic bacteriology offer two distinct atmosphere generation options. The first option is an anaerobic environment, which provides an oxygen-free atmosphere with a concentration of 0.2% O_2_ or less. The second option provides a concentration of 5% O_2_ for microaerophilic conditions. Following the R&D phases, our system was also optimized to cultivate the specified bacterial species.

**Figure 3:**
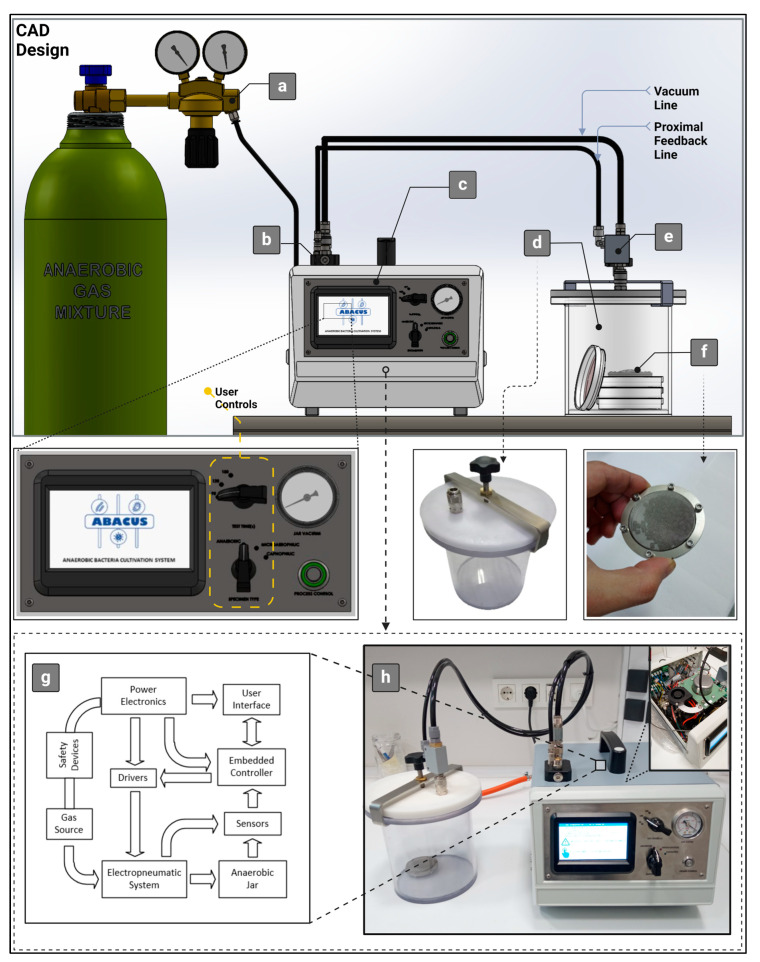
The schematic representation of ABACUS system: (**a**) pressurized gas supply, (**b**) connection manifold, (**c**) control unit and user interface, (**d**) anaerobic jar and lid clamp, (**e**) jar socket, (**f**) catalyst sachet, (**g**) block diagram, (**h**) device under test.

We have referred to our system as the ABACUS (Anaerobic/Microaerophilic Bacteria Cultivation System). The detailed overview of system components can be seen in Figure 3. The control unit forms the core of the ABACUS system. The control unit provides an interface for the user to run the gas exchange, select atmospheric options, and test periods (90, 120, or 180 s), and also contains digital and analog process indicators. The user inputs collected from this interface are processed by a microcontroller-based (STM32F446RE ARM-Cortex MCU, ST Microelectronics, Geneva, Switzerland) embedded control board. All measurements, calculations, and operational steps related to the ongoing atmosphere generation process are displayed on the TFT screen (4.3 inch—480 × 272, SATOZ). In order to monitor the source pressure and jar vacuum, a number of pressure sensors ((9000405, Metal Work Pneumatic, Arlington, TX, USA), (HSC series ± 100 mbar Board Mount, Honeywell, Charlotte, NC, USA), (BCT22 1/8 NPT, ATEK Sensor, Kocaeli, Turkey)) were integrated into the control unit. The measurement accuracy of these sensors was validated by a calibration bench (RT304 Calibration Trainer, G.U.N.T. Gerätebau GmbH, Barsbüttel, Germany). Due to the power and control requirements of system components, a custom-made power management board and driver board were designed and fabricated. An automotive-grade dry vacuum pump (rotary vane, 12V DC, 150 W) was modified to achieve the specific vacuum requirements of the ABACUS control unit and used to evacuate the gas content of the jar. To manage the gas exchange cycles, several 3/2 solenoid valves were utilized. Additionally, a pressure relief valve was added to the pneumatic system to prevent overpressure conditions during a systematic breakdown scenario. Electronic and pneumatic system components are outlined via a simple block diagram, given in Figure 3g. The mechanical parts (Figure 3b–d), including the anaerobic jar (lid and jar body), manifolds, and TFT front panel, were designed using CAD software (SolidWorks^®^, Dassault Systemes, 2015) and manufactured by a CNC machine using engineering plastics (e.g., polycarbonate, Delrin). The conventional pneumatic quick connectors equipped with check valves were placed on the jar lid and connection manifold. These types of connectors were selected intentionally to isolate the jar atmosphere from oxygen-rich air during disconnection from the ABACUS. Also, to guarantee air tightness, an O-ring seal was installed on the jar lid. The lid clamp apparatus used to secure the jar atmosphere was manufactured from laser-cut sheet metal and bent. The catalyst sachet (Figure 3f) was assembled from laser-cut metal rings and stainless steel mesh. The control and analysis algorithms were coded using the C and C++ programming languages and integrated into the embedded system. Further details about the software can be found in the proposed method section. Figure 3h demonstrates the actual system in operation.

#### 3.1.2. Clinical Validation of Basic System Features

The presented system provides two fundamental atmospheric options for microbial cultivation: anaerobic and microaerophilic. A series of cultivation experiments were conducted to validate the atmospheric features of the designed cultivation system. During the planning stages of the experiments, the candidate bacterial strains were selected from obligate anaerobes intentionally. The major motivation underlying this approach is the creation of a worst-case scenario to test the cultivation capability of the ABACUS system. Because obligate anaerobes are the species that need strictly anaerobic conditions to grow, the cultivation performance of the jar gassing system under these circumstances can be regarded as a reliable indicator of efficiency. In order to provide a comparative performance assessment, bacteria-inoculated media were simultaneously cultivated via ABACUS and a conventional anaerobic gas pouch.

The standard American Type Culture Collection (ATCC) strains selected for the performance tests were as follows: *Bacteroides fragilis* (ATCC^®^ 25285), *Prevotella bivia* (ATCC^®^ 29303), *Cutibacterium acnes* (ATCC^®^ 29399), *Peptoniphilus asaccharolyticus* (ATCC^®^ 29743), (Finegoldia magna ATCC^®^ 29328), *Parvimonas micra* (ATCC^®^ 33270), *Campylobacter jejuni* (ATCC^®^ 33560). These specific organisms were preferred due to their classification as pathogens with high disease-causing potential and their presence as stock species within the cold chain of the anaerobic bacteriology laboratory, where the clinical experiments were conducted. Following the broth cultivation of the strains, the bacterial suspensions (approximately 1 × 10^8^ CFU mL^−1^) were subcultured on agar plate couples (Brucella Agar, CM0271, Oxoid, supplemented with appropriate ingredients such as Vitamin K_1_ (phytomenadione) and Hemin (from bovine, ≥90%, H9039, Sigma-Aldrich, St. Louis, MO, USA)). The first series of agar plates were incubated at 37 °C in anaerobic conditions generated by the ABACUS system. During the cultivation of the first series of agar plates, steel catalyst sachets that contain 12 g of pellets (Deoxo D, 0.5% Pd, Anco Catalysts, Bromsgrove, UK) were placed into jars to remove the residual oxygen. For the second series of inoculated agar plates, an anaerobic atmosphere was generated using the GENBox disposable sachets (bioMerieux^®^, Marcy Etoile, France) and the plates were incubated at 37 °C for 48 h. At the incubation stages, an anaerobic test strip covered with a redox-sensitive dye (Anaerotest™ Strips, 1323710001, Sigma-Aldrich) was used to verify that the anaerobic atmosphere of the jars was maintained. Freshly opened strips were placed in each jar.

In addition, a disk diffusion test was performed to indicate the viability of the cultured microorganisms through inhibition zones. For this purpose, different types of antibiotic discs (such as ciprofloxacin, azithromycin, clindamycin, gentamicin, metronidazole, or tetracycline from Oxoid) were selected and placed on the surface of agar plates containing the *Campylobacter jejuni* strain. The microaerophilic atmosphere generation option of the ABACUS device was used to cultivate this bacterium.

#### 3.1.3. Gas Exchange Model of the Anaerobic Jar Atmosphere

The control unit of the ABACUS system uses a series of mathematical expressions based on the ideal gas law to calculate the concentration changes that occur in the gaseous atmosphere of the jar during the evacuation and replacement cycles. The only feedback channels between the jar environment and the control unit are the vacuum pressure and proximal line. Therefore, all mathematical descriptions run in the software were formulated based on pressure measurements. In contrast to conventional systems, the ABACUS system contains an auxiliary pressure line to prevent measurement errors originating from gas flow and pipe length.

The gas concentrations in the jar were defined in mass percentages and represented by a vector. *γ_Jar_* is a vector containing gas concentrations, $\gamma_{Jar}=\left[\gamma_{O_{2}}\;\gamma_{N_{2}}\;\gamma_{Ar}\;\gamma_{H_{2}}\;\gamma_{{CO}_{2}}\right]$, and updated after each gas exchange cycle. A different concentration vector was defined for the anaerobic gas mixture that was used for replacement; it is given as $\gamma_{Cylinder}=\left[\gamma_{O_{2}}\;\gamma_{N_{2}}\;\gamma_{Ar}\;\gamma_{H_{2}}\;\gamma_{{CO}_{2}}\right]$. Although the anaerobic gas mixture does not contain oxygen or argon, the size of the vector is defined as equal for algorithmic reasons, and percentages of these gases are counted as zero for *γ_Cylinder_*. The molar mass of gases was also specified in vector form, $M_{Gases}=\left[M_{O_{2}}\;M_{N_{2}}\;M_{Ar}\;M_{H_{2}}\;M_{{CO}_{2}}\right]$. The concentration changes in the anaerobic jar were modeled using the following equations:

(2) $$m_{{Jar}_{i}}\left(\theta +1\right)=\left(\frac{\left(P_{Atm}-P_{Vac}\right)\cdot V_{Jar}}{R\cdot T}\right)\cdot \gamma_{{Jar}_{i}}\left(\theta \right)\cdot M_{{Gases}_{i}}$$

(3) $$m_{Jar}\left(\theta +1\right)=\sum_{i=1}^{k}m_{{Jar}_{i}}\left(\theta +1\right)$$

(4) $$\Delta P_{Jar}=P_{Atm}-P_{Vac}$$

(5) $$m_{{Cylinder}_{i}}=\left(\frac{\left(P_{Atm}-{\Delta P}_{Jar}\right)\cdot V_{Jar}}{R\cdot T}\right)\cdot \gamma_{{Cylinder}_{i}}\cdot M_{{Gases}_{i}}$$

(6) $$m_{Cylinder}=\sum_{i=1}^{k}m_{{Cylinder}_{i}}$$

(7) $$m_{Total}\left(\theta +1\right)=m_{Jar}\left(\theta +1\right)+m_{Cylinder}$$

(8) $$\gamma_{{Jar}_{i}}\left(\theta +1\right)=\frac{m_{{Jar}_{i}}\left(\theta +1\right)+m_{{Cyclinder}_{i}}}{m_{Total}\left(\theta +1\right)}$$

where *θ* and *θ* +1 are the current and next gas exchange cycles, respectively, *i* is the index of the concentration and molar mass vectors, *m* represents the mass of in and out gases, *P_Atm_* stands for atmospheric pressure, *P_Vac_* is the vacuum pressure, Δ*P_Jar_* is the pressure change within the jar, *V_Jar_* denotes jar volume, *R* and *T* represent the molar gas constant and temperature of the jar environment, respectively. In the calculations, all pressure values were expressed as absolute pressure. The initial values of the jar concentration vector were determined due to the GC/MS measurements of atmospheric gas mixture. The impact of dead volumes in pipes on the calculation is not considered significant and therefore not included.

### 3.2. Microkinetic Modeling of Pd/Al_2_O_3_ Catalyst

Throughout the design and experimental phases, observations consistently indicated the deterministic impact of the palladium catalyst on the evacuation and replacement process. Therefore, in order to build a better model and obtain effective estimation results, we decided to examine the palladium catalyst from a comprehensive perspective. For these purposes, the catalysts used in the ABACUS system were initially studied in the context of material science and surface chemistry. Afterwards, the surface reaction that occurred in the jar was modeled using a computational chemistry approach.

#### 3.2.1. Characterization of Palladium Catalyst

Heterogeneous catalysts doped with palladium or other noble metals have been used in numerous processes in the chemical industry for many years [38]. In the scope of anaerobic bacteriology, they are frequently used as deoxygenation catalysts. The definition of heterogeneous is fundamentally related to the surface interface where the reaction occurred. As in our study, different gas species bind to each other on a solid surface that is provided by the catalyst via the reducing effect of the transition metal. These phase differences between the reactants and catalyst structure form heterogeneity. In the nanoscale world of surface reactions, the gas or liquid species are interacting with each other on the vacant sites. The empty sites can be seen as impurities in the crystal structures. These defects are also named active sites, and they constitute a very small portion of the catalyst body. To create such surfaces, a binary crystal structure used as a supporting material is processed with a noble metal. The support material also provides a large surface area to increase the production rate via its porous structure [39].

The most commonly used heterogeneous catalyst combination in evacuation and replacement systems is Pd/Al_2_O_3_. This type of catalyst allows the generation of oxygen and hydrogen recombination reactions at relatively low (e.g., room or body) temperatures. A detailed description related to these reactions is given in the following sections. In our study, 0.5% doped alumina pellets were used for oxygen gas removal. The material information obtained from the specs provided by the manufacturer (Anco Catalysts) is given in Table 2.

Several material analyses were performed and interpreted to validate these specifications and to obtain additional chemical and morphological data for modeling. During all characterization steps, freshly baked catalyst pellets were examined.

##### Scanning Electron Microscopy (SEM) Analysis

The morphological examination based on the electron microscopy is a very sophisticated and tremendously utilized method in the surface science field. SEM also provides invaluable information on the surface structure of the samples under investigation, with a resolution at the nanometer scale. The morphological investigation of catalyst pellets was performed using a ZEISS EVO MA 10 scanning electron microscope (Jena, Germany) with an energy-dispersive X-Ray spectroscopy (EDS) detector. This detector allows the elemental analysis of the observed surfaces. Prior to imaging, the catalyst pellets are coated with a Au/Pd mixture to prevent the accumulation of electrons on the surfaces. A Quorum SC7620 sputter coater was used for the coating. In order to minimize EDS error associated with the palladium content of the cover material, the coating time and current were selected from relatively low values (30 s and 18 mA).

##### X-Ray Diffraction (XRD) Analysis

The X-Ray diffraction analysis is a non-destructive analysis method widely used in the identification of the crystal structure of materials. Some crystal facets of catalysts provide a lower energy barrier to the reaction intermediates than the other facets [40]. In catalyst science, the determination of these favorite crystal surfaces has significant importance for the characterization of the dynamic behavior of solid–gas interactions and the calculation of reaction model parameters [41].

The diffraction peaks indicating the crystal structure of the catalyst were obtained using a SHIMADZU XRD-6100 X-Ray diffractometer (Nakagyo-ku, Kyoto, Japan) The XRD-6100 was equipped with a CuKα radiation (λ = 0.15406 nm) target. The diffraction signals were collected between 5° and 80° with a sampling pitch of 0.02. Measurements were performed under conditions of 40 kV voltage and 30 mA current.

#### 3.2.2. Gas Chromatography–Mass Spectrometry (GC/MS) Analysis

The concentrations of atmospheric gases in the cultivation environment and the concentration of the anaerobic gas mixture used for replacement are crucial factors that must be taken into account when performing estimation calculations. In order to obtain reliable input parameters for the simulation experiments, a number of GC/MS analyses were performed. A miniature pressure container equipped with a flow control valve was fabricated to transport the gas samples. The GC/MS analysis is a robust analytical test method used to identify the concentration of liquid or gas mixtures. The Agilent 6890N network gas chromatograph (Santa Clara, CA, USA) was used during the measurements. The set-up parameters of the instrument are as follows: carrier gas (Ar) flow rate: 20 mL/min, detector temperature: 250 °C, Owen temperature: 250 °C, injection gas volume: 1 mL. The device was calibrated with a special gas mixture (1.02% H_2_, 5.13% CO_2_, 10.25% O_2_, N_2_ (Balance)) under constant gas flow (0.5 mL min^−1^) before the analysis. Two different gas mixtures were analyzed to identify the input conditions. The results of the chromatography measurements are given in Table 3.

#### 3.2.3. Modelling Methodology

From a structural perspective, anaerobic jar gassing systems can be viewed as a type of fixed-bed catalytic reactor system without continuous reactant flow. Under controlled conditions (e.g., temperature, pressure, and reactant concentrations), in such a reactor system, the most significant factor that influences the production rates is the heterogeneous catalyst. In order to estimate the catalyst behavior and the surface reaction that occurred, a multiscale modeling approach is typically used, which combines computational and experimental findings [42]. This methodology covers a wide range of examination stages, starting from the nanoscale (electronic structure and molecular dynamics) to the macroscale (CFD) [43,44]. The following sections present the details of our data-driven and multiscale modeling approach.

##### Surface Reaction Scheme

In a surface reaction occurring on a catalyst, the reaction products are formed through the completion of several elementary reaction steps. At the very beginning of the reactions, the gaseous species (O_2_ and H_2_ in our case) in the reactor adsorb to vacant sites on the crystal structure of the catalyst. Subsequently, these surface species interact with each other according to the minimum energy path of the elementary reaction step. Finally, the produced molecule is immediately desorbed from the catalyst surface. This surface reaction mechanism is one of the most utilized reaction mechanisms in surface chemistry and is referred to as the Langmuir–Hinshelwood mechanism [45]. The elementary reaction steps of H_2_O formation from H_2_ and O_2_ over palladium surfaces, in accordance with Langmuir’s mechanism, are outlined in Equations (9)–(14).

(9) $$O_{2}+2*\;\;\to \;2\;O^{*}$$

(10) $$H_{2}+2*\;\;⇋\;2{\;H}^{*}$$

(11) $$H^{*}+O^{*}\;\;⇋\;\;{OH}^{*}+*$$

(12) $$H^{*}+{OH}^{*}\;\;⇋\;\;H_{2}O^{*}+*$$

(13) $${2OH}^{*}\;\;⇋\;\;H_{2}O^{*}+O^{*}$$

(14) $$H_{2}O^{*}\;\;\to \;\;H_{2}O\;+*$$

The aforementioned reaction scheme was selected from computational chemistry studies that focused on hydrogen oxidation reaction on platinum-group metal surfaces [46,47]. The first and second reaction steps depict the dissociative adsorption of gas species. The remaining reaction steps demonstrate the interactions of weakly bonded surface atoms with metal atoms. The asterisk denotes vacant sites on the catalyst surface. The microkinetic model calculations were performed based on these reaction steps.

##### DFT (Density Functional Theory) Calculations

**Figure 4:**
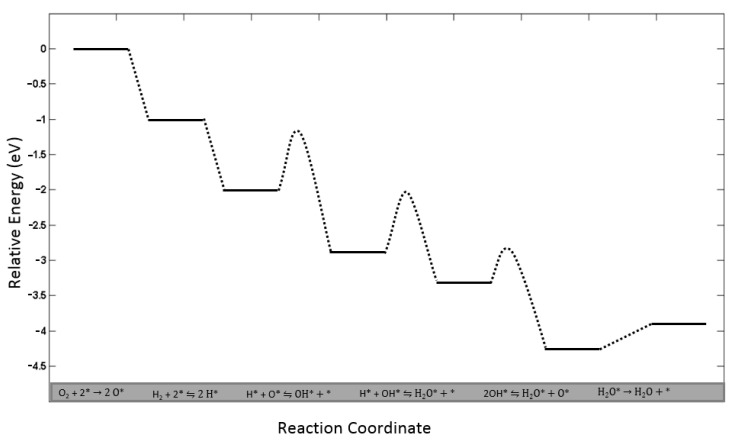
Energy diagram of H_2_O formation steps over palladium catalyst. Reconstructed from Ref. [46].

Density functional theory calculations provide a comprehensive insight into the structure of catalysts at the atomistic level. In the study of heterogeneous catalysis, DFT simulations are often combined with experimental results to build more realistic models. In the present study, certain microkinetic model parameters of elementary reactions that are described via transition state theory were obtained from DFT studies that are documented in the chemical physics literature. The numerical data presented in these studies were confirmed through thermodynamic evaluation and adapted. Dietze and coworkers’ study [46] that focused on water formation on Pd(111) surfaces was taken as a reference in this context. In order to derive the pre-exponential factor of the elementary reaction steps outlined in the previous section, the vibrational entropy values of surface reactions were calculated using the vibrational frequencies of reaction intermediates provided by this research. The energy diagram of elementary reactions (Equations (9)–(14)) is depicted in Figure 4.

The pre-exponential factor of forward reaction rates (*β_f_*) was calculated by Equation (15). In this expression, *k*_B_ and *T* are the Boltzmann constant and the reaction temperature, respectively, and *h* denotes Planck constant. The key parameter linked to molecular dynamics is the vibrational entropy (${\Delta S}_{f}^{o‡}$) of the associated reaction step.

(15) $$\beta_{f}=\frac{k_{B}\cdot T}{h}\cdot exp\left(\frac{{\Delta S}_{f}^{o‡}}{k_{B}}\right)$$

The vibrational modes constitute the major portion of the entropy contribution for the adsorbed surface intermediate. Therefore, the entropy statement ${\Delta S}_{f}^{o‡}=S_{{AB}^{‡}}^{o}-\left(S_{A^{*}}^{o}+S_{B^{*}}^{o}\right)$ was calculated using vibrational entropies of the transition state ($S_{{AB}^{‡}}^{O}$) and the reactants ($S_{A^{*}}^{o}\;,\;S_{B^{*}}^{o}$), where the entropy definitions belong to an elementary reaction that is given as $A^{*\;}+B^{*}⇄\;{AB}^{*}$. In surface chemistry, the vibrational frequencies provided by DFT or experiments are treated frequently with the harmonic normal mode approximation to determine the entropies of the surface species. The approximation statement used in the study is given by:

(16) $$S_{vib}=k_{B}\cdot \sum_{i}^{N_{mode}}\left(\frac{x_{i}}{exp\left(x_{i}\right)-1}-\ln\left(1-exp\left(-x_{i}\right)\right)\right)$$

where *x_i_* represents the entropy contribution of each vibrational mode, and this parameter is expressed as:

(17) $$x_{i}=\frac{h\cdot v_{i}}{k_{B}\cdot T}$$

where *v_i_* denotes the vibrational frequencies of the surface intermediates, *N_mode_* stands for the number of vibrational modes, *i* is the index of the vibrational modes defined by $3N_{i}-5$ (Linear molecules) and $3N_{i}-6$ (Nonlinear molecules) expressions.

##### Microkinetic Modeling Details

The microkinetics simulations were conducted using the MKMCCX software (version 1.54) developed by Filot and colleagues [48]. Individual reaction rate constants were calculated for each elementary reaction step, and the overall reaction behavior was analyzed. The elementary reactions involving adsorption and desorption processes (Equations (9) and (10)) were modeled with collision theory based Hertz–Knudsen-type equations [49]. The rate constants for the adsorption (*k_ads_*) of diatomic gases were calculated using the following equation:

(18) $$k_{ads}=\frac{A_{site}\cdot S}{\sqrt{2\cdot \pi \cdot m\cdot k_{B}\cdot T}}\cdot exp\left(-\frac{E_{ads}}{k_{B}\cdot T}\right)$$

where *S* and *A_site_* are the sticking coefficient and surface site area, respectively, *k*_B_ is the Boltzmann constant, *T* denotes the reaction temperature, *m* is the mass of the molecule, *E_ads_* denotes the adsorption energy of the molecule. In the relevant literature, the adsorption processes are usually reported as a non-activated step. Therefore, the exponential term is often not considered [50]. For a diatomic gas molecule, the rate of desorption is given by:

(19) $$k_{des}=\frac{k_{B}\cdot T^{3}}{h^{3}}\cdot \frac{A_{site}\cdot \left(2\cdot \pi \cdot m\cdot k_{B}\right)}{\sigma \cdot \theta_{rot}}\cdot exp\left(-\frac{E_{des}}{k_{B}\cdot T}\right)$$

where *k_des_* is the rate of reaction (desorption), *σ* and *θ_rot_* are the rotational symmetry factor and rotational temperature, respectively, *h* stands for the Planck constant, *E_des_* denotes the desorption energy of molecule [51]. To calculate the surface site area, Equation (20) was used [52]. With a value of 10^−15^ cm^2^ per site for *A_site_*, it can be employed in practical calculations [53], where *L* represents crystallite size calculated from XRD measurements, *d* and *m* are the atomic radius and atomic mass of palladium, respectively, *ρ* denotes the bulk density of the metal atom, and *N_Av_* is the Avogadro number.

(20) $$A_{site}=0.6\cdot \left(\frac{d\cdot m}{\rho \cdot N_{Av}\cdot L}\right)$$

The desorption energies used in the calculations have been collected from a variety of studies that have focused on surface reactions over palladium catalysts [54,55]. To determine the forward and backward rates of surface reactions (Equations (11)–(14)), a transition state theory (TST)-based relation was used. The rate definition is as follows:

(21) $$k_{f,b}=\beta_{f,b}\cdot exp\left(-\frac{E_{f,b}}{R\cdot T}\right)$$

where the letters *f* and *b* denote forward and backward reactions, *β* is the pre-exponential factor (or frequency factor), *E* represents the activation barrier height of an elementary reaction, *R* is the molar gas constant, and *T* denotes the reaction temperature. In order to guarantee equilibrium conditions for the forward and backward reaction rates, the microkinetic model calculation was performed based on a thermodynamic equilibrium constant, where *K_equ_*, the equilibrium constant, is given as:

(22) $$K_{equ}=\frac{k_{f}}{k_{b}}$$

Furthermore, the equilibrium constant defines the relationship between the Gibbs free energy of reactions and the reaction entropy and enthalpy [56]. *K_equ_* can be written as:

(23) $$K_{equ}=exp\left(\frac{-\Delta G{}^{\circ}}{k_{B}\cdot T}\right)=exp\left(\frac{\Delta S{}^{\circ}}{k_{B}}\right)\cdot exp\left(\frac{-\Delta H{}^{\circ}}{k_{B}\cdot T}\right)$$

where Δ*G*°, Δ*S*° and Δ*H*° represent the changes in standard Gibb’s free energy, entropy, and enthalpy, respectively. The remaining simulation parameters, including gas concentrations (via partial pressures) and temperature ranges (18–37 °C), were determined through the mathematical model of the gas exchange cycles (see Section 3.1.3) managed by the ABACUS.

### 3.3. Proposed Estimation Algorithm

To estimate the residual oxygen content of the anaerobic jar, a hybrid approach was performed. The first step of this approach is the microkinetic modeling of the palladium catalyst behavior based on a first-principles calculation; the second step is the minimization of the reaction rate differences between the model and the real system outputs. To close this gap, the parameters of the rate equation were tuned using a non-model-based real-time optimization approach. The numerical optimization was performed through a discrete form of a deterministic extremum seeking algorithm. Afterwards, the tuned equation was used for the estimation. The extremum seeking algorithm is a variant of the gradient descent optimization method. Fundamentally, it uses a sinusoidal perturbation signal to manipulate the system variables and simultaneously observe the system response. Following the interpretation of these observations, the algorithm tries to identify the optimal levels of parameters. Frequently, tuning ratios of parameters are determined via a cost function [57].

**Figure 5:**
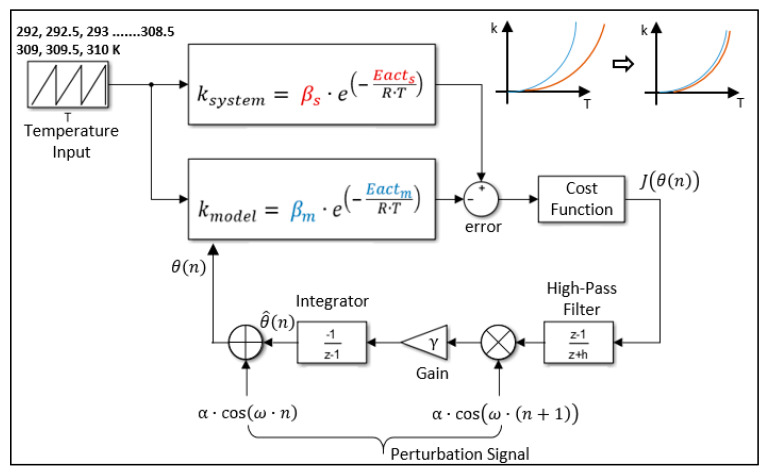
Extremum seeking optimization scheme.

As previously outlined in the modeling section, the Langmuir–Hinshelwood surface reaction mechanism consists of several elementary reaction steps. One of these steps is referred to as the rate-determining step (RDS). The RDS is a dominant reaction step and can be used to represent the overall behavior of the catalytic reaction. The reaction rate of the RDS was defined by an Arrhenius-type equation. The two main coefficients of this statement are presented in vector form. *θ* is the coefficient vector, $\theta ={\left[E_{act},\;\beta \right]}^{T}$. The expressions used for the numerical optimization of Arrhenius equation coefficients adapted from refs. [58,59] are given as:

(24) $$\xi \left(n\right)=-h\cdot \xi \left(n-1\right)+J\left(n-1\right)$$

(25) $$\;\hat{\theta_{t}}\left(n+1\right)=\hat{\theta_{t}}\left(n\right)-\gamma_{t}\cdot \alpha_{t}\cdot \cos\left(\omega_{t}\cdot n\right)\cdot \left[J\left(n\right)-\left(1+h\right)\cdot \xi \left(n\right)\right]$$

(26) $$\theta_{t}\left(n+1\right)=\hat{\theta_{t}}\left(n+1\right)+\alpha_{t}\cdot \cos\left(\omega_{t}\cdot \left(n+1\right)\right)$$

where *t* represents the index of the coefficient vector, *n* is the iteration number, *ξ* is the bias level, *J*(*n*) indicates the error value of the *n*th iteration. *θ* denotes the optimized value for each parameter, *ω* is the perturbation frequency (rad s^−1^), *γ* and *α* are the estimation gain and perturbation step size, respectively, and *h* stands for the high-pass filter denominator. The extremum seeking-based optimization scheme is illustrated in Figure 5.

It is important to consider some limitations during the identification of modulation frequency and filter coefficient to ensure robust optimization. The modulation frequency should be determined according to the expression $\omega =a^{t}\cdot \pi$, wherein $0<\left|a\right|<1$. The high-pass filter should be defined within the 0<h<1 range. In order to facilitate the implementation of the extremum seeking algorithm in a microcontroller-based digital system, a discrete form of the algorithm was utilized. The Arrhenius-type relation signifies the temperature dependence of the reaction rate; therefore, an iterative temperature input was applied with a resolution of 0.5 Kelvin to draw k-T graphs. These response curves were used to calculate the error term. This parameter is also used as an input for the cost function, which is employed to evaluate the convergence efficiency of the algorithm. A root mean square error definition was selected as the cost function, which is defined as follows:

(27) $$J\left(n\right)=\sqrt{\sum_{i=1}^{N}\frac{{\left(k_{system}-k_{model}\right)}^{2}}{N}}$$

where *N* denotes the size of the temperature input array, and *J*(*n*) is the calculated cost value for each iteration. The extremum seeking method is an iterative optimization technique. The iterations were terminated when the root mean square error of the optimization process reached the predefined termination criteria (approx. 10^−4^).

The ABACUS control unit performs a series of quality assurance tests to guarantee the jar sealing and catalytic activity of the Pd/Al_2_O_3_ sachet are at an acceptable level. These test steps are referred to as leakage and catalyst tests, respectively. The duration of the test steps is selected from the user interface before the gas exchange process starts. During the catalyst test, the jar is filled with a certain amount of anaerobic gas mixture, allowing the recombination reaction to occur. The pressure loss observed during the catalyst test can be regarded as an indirect indicator of the quantity of H_2_O formed within the jar. Based on this measurement, the molar fraction of H_2_O (Δ*n*) produced over palladium was calculated via Equation (28). The rate expression of the catalytic reaction (*k_system_*) was obtained by calculating the turning over frequency (*TOF*, mol s^−1^) with the associated pressure loss (Δ*P_loss_*), catalyst test time (Δ*t_cat_test_*), and temperature of the clinical environment (Equation (29)). The TOF value is a measure of catalytic activity used in heterogeneous chemistry.

(28) $$\Delta n=\frac{{\Delta P}_{loss}\;\cdot \;V_{Jar}}{R\cdot T}$$

(29) $$TOF=\frac{\Delta n}{{\Delta t}_{cat\_test}}$$

The derived relationships between the actual catalytic behavior of the system and the Arrhenius expressions are illustrated in Equations (30) and (31). The linear form of expression was used to demonstrate the rate constant.

(30) $$\ln\left({TOF}_{@T_{tof}}\right)=\left[\left(-\frac{E_{{act}_{s}}}{R}\cdot \frac{1}{T_{tof}}\right)+\ln\left(\beta_{s}\right)\right]$$

(31) $$\ln\left(k_{system}\right)=\left[\left(\frac{\ln\left({k_{s}}_{@292K}\right)-\ln\left({TOF}_{{@T}_{tof}}\right)}{\frac{1}{292}-\frac{1}{T_{tof}}}\cdot \frac{1}{T}\right)+\ln\left(\frac{k_{B}\cdot T_{tof}}{h}\right)\right]$$

where *T_tof_* represents the specific temperature value that was recorded at the time the TOF measurements were taken, and *k_s@_*_292K_ is the predefined reaction rate coefficient obtained from experiments conducted at 292 K.

The main objective of our proposed methodology is to enhance a reaction model constructed from material characterization and molecular simulations with the guidance of real system outputs, thus developing a more reliable behavioral model that can be used to predict the gaseous atmosphere of the jar.

## 4. Results and Discussion

The findings were elucidated in four main topics: results of clinical validation tests, characterization results, the microkinetic model outputs and analysis of the extremum seeking algorithm.

### 4.1. Results of Clinical Validation Tests

Following the completion of the incubation phase, plates were examined in a culture-dependent framework. The viability assessment of the culture plates was performed via plate counting and morphological evaluation. The other conventional and culture-independent methods used in clinical microbiology for determining cell viability were not employed during the experiments. Detailed clinical examinations (e.g., identification of an unknown species in a pathological sample) that relate to bacterial species are considered beyond the scope of this study.

**Figure 6:**
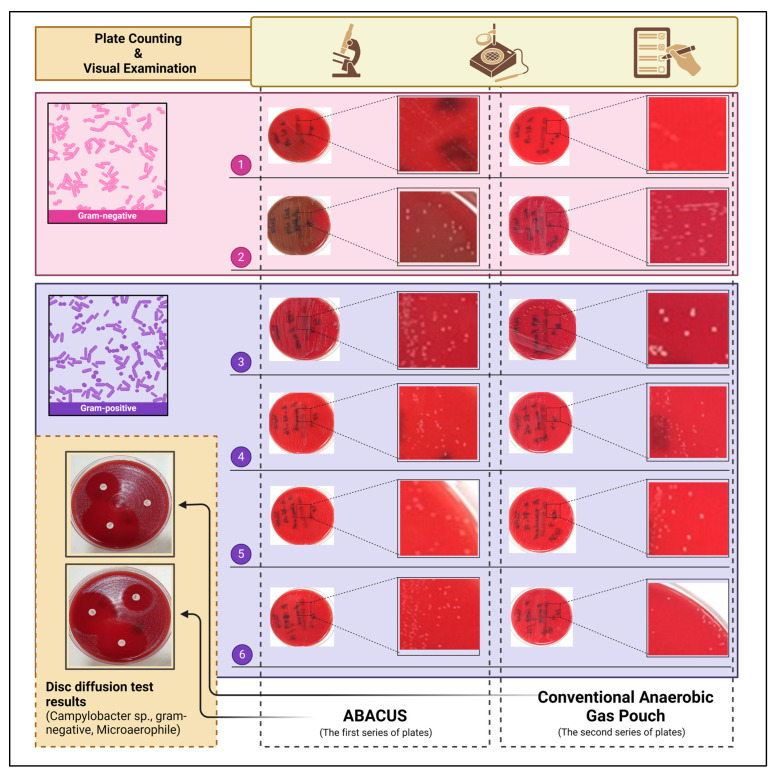
The schematic representation of cultivated bacterial colonies via two different systems. Gram-negative (1 to 2) and Gram-positive (3 to 6) bacteria are illustrated in a separate section. The specific details of the bacterial species are given in Table 4.

From a morphological perspective, the results shown in Figure 6 clearly demonstrate that all selected bacterial strains presented prominent growth on the culture medium. Furthermore, there is no indication of contamination. Additionally, these colonial formations constituted a reliable basis for plate counting. The disc diffusion test provides an additional metric for evaluating the cultivation performance of both systems. The inhibition zones created by colonies can be interpreted as an indicator of viable cell activity. This test was also performed for the validation of the microaerophilic atmosphere generation feature. As can be seen from the numerical results given in Table 4, the number of colonies cultivated by ABACUS is considerably close to the conventional anaerobic atmosphere creation sachet. Nevertheless, previous clinical studies that focused on anaerobic cultivation methods have revealed that evacuation and replacement devices enable denser colonial growth during the cultivation of obligate anaerobes [60]. From this perspective, the variability observed in the ABACUS results was not considered a dysfunctionality. In addition, it was concluded that the variance in colony counts was dependent on the palladium catalyst performance.

Consequently, it can be stated with certainty that the ABACUS system is capable of creating anaerobic and microaerophilic conditions at the conventional laboratory level, as demonstrated by the results of the clinical experiments.

### 4.2. Characterization Results

#### 4.2.1. SEM Findings

**Figure 7:**
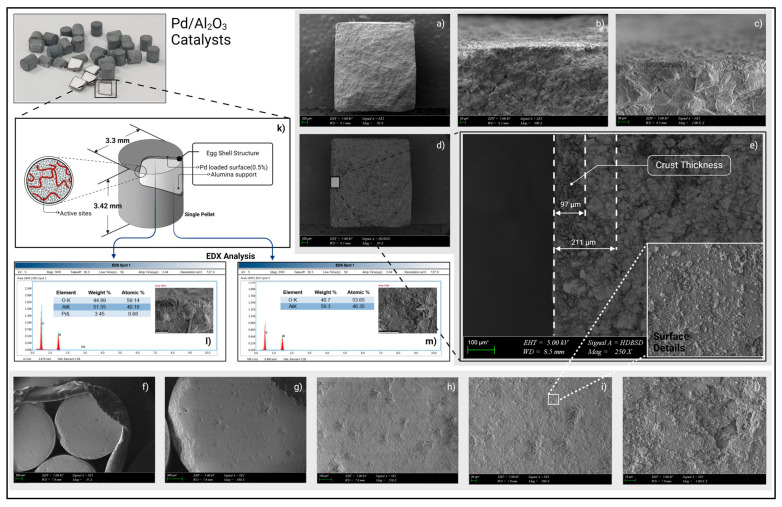
The scanning electron microscopy images (EHT = 5kV). Cross-sectional views: SEI detector images (**a**–**c**), BSD detector images (**d**,**e**). Top views: SEI-based images (**f**–**j**), Mag: 45×, 100×, 250×, 500×, and 2000×, respectively. Illustration of eggshell structure (**k**). EDX measurements of shell and support material (**l**,**m**).

A detailed overview of SEM images is given in Figure 7. The images were collected from two different surfaces of pellets. The top surface and cross-sectional surface examinations clearly showed the porous structure of the pellets. At higher magnifications, the particle size differences of the support material and doped metal can be observed with greater ease (e.g., Figure 7c,j). During the cross-sectional examinations, ordinary surface images were obtained with a secondary electron (SEI) detector. To enhance the contrast between the catalyst and body materials, a back-scattered electron (BSD) detector was used. The images produced by the BSD detector can reveal the density variations in the materials. The palladium shell thickness was measured from the BSD images using *ImageJ* software(version 1.54). This thickness parameter can be regarded as a sign of an active catalyst. The crust thickness values (Figure 7e) obtained were found to be consistent with values reported in the literature [61,62]. Several EDS analyses were performed to validate the surface and body contents of the pellets. The palladium content of the shell and the alumina content of the support material were confirmed through the EDS analysis.

#### 4.2.2. XRD Findings

**Figure 8:**
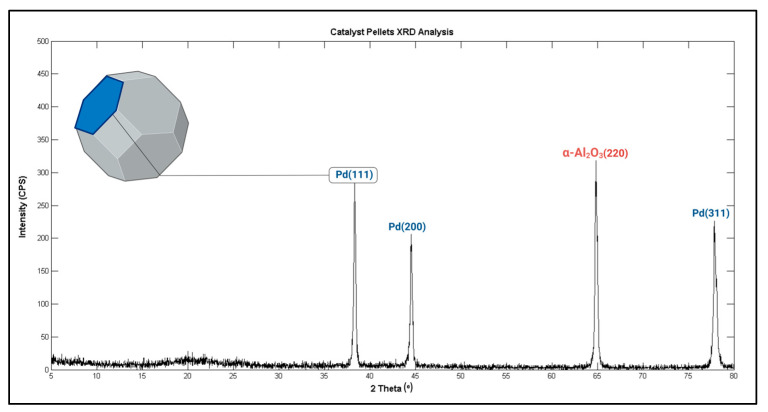
The diffraction pattern of palladium catalyst pellets.

Figure 8 shows recorded XRD peaks. These diffraction results indicate four different reflection planes or facets. The miller indexes of these reflection planes were identified from related scientific studies that include ICDD (The International Centre for Diffraction Data, JCPDS card, file no.46-1043) reference peaks of palladium [63]. Numerous studies have shown that the oxygen and hydrogen recombination reactions that occur on palladium surfaces are mostly generated on Pd(111) facets [64,65,66,67]. The favorable facets of reactions determined from X-Ray diffractions are a major simulation parameter for microkinetic modeling. The needle-like shapes of XRD peaks also demonstrate a well-ordered crystal structure rather than an amorphous structure [68,69]. It can be considered as indicative of the fact that the pellets do not have any fatigue composition. Another finding that can be stated from the diffraction patterns is the peak of the support materials, which indicates the α-form of alumina. The detailed surface image in the SEM results given in Figure 7i also supports this claim. The final data that can be obtained from diffraction analysis is the crystallite size of each plane. The most widely used equation to calculate crystallite size is the Debye–Scherrer formula [70]. This equation is given Equation (32). In this equation, *β* is the full width at half maximum (FHWM) value in degrees, *θ* is the 2Theta angle of the peaks, *K* is the Scherrer shape factor and *λ* is the wavelength of the target. The calculated sizes can be seen in Table 5.

(32) L=Kλβ cos⁡θ

Crystallite size is an important factor that affects catalyst activity and selectivity. Activity and selectivity may vary due to metal doping concentration and surface area [71,72,73]. Nevertheless, literature findings indicate that nanoscale crystallite size is a sign of catalytically active facets [66,74].

The results of the XRD analysis elucidated that the palladium pellets under examination are catalytically active, and their Pd(111) plane must be used for modeling purposes.

### 4.3. Microkinetic Model Outputs

The calculated parameters for the microkinetic modeling of the hydrogen oxidation reaction over Pd(111), as referenced in the section entitled DFT (Density Functional Theory) Calculations and Microkinetic Modeling Details, are presented in Table 6 and Table 7. The pre-exponential factors of forward reactions were defined via vibrational frequencies of surface species and transition states. For backward reactions, these values were calculated with activation barrier heights and entropy values using the thermodynamic equilibrium expression (Equation (23)). The model parameters obtained are consistent with the data presented in the reviewed studies within the relevant literature [47,75].

The desorption energies of the reactions defined by the collusion theory were taken as 0.8 eV and 1.0 eV for Equations (9) and (10), respectively. The sticky coefficient was taken as 1.0 and 0.5 for stoichiometric reasons. Rotational temperature and symmetry factors were chosen from molecular chemistry books, and the values are consistent for diatomic and homonuclear molecules. The value of the active site area was calculated from the XRD results.

**Figure 9:**
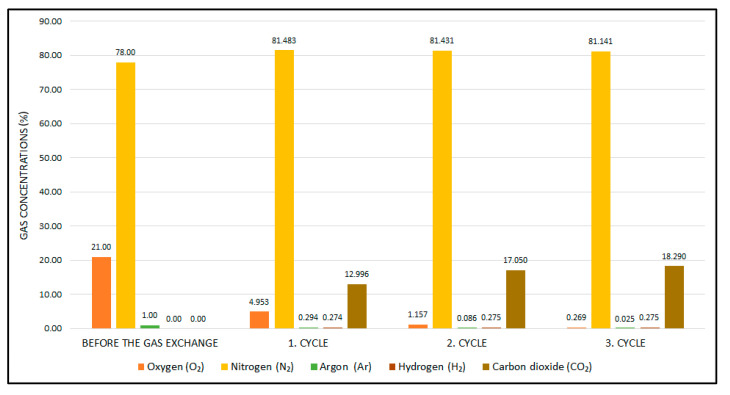
Concentration variations.

Another crucial model parameter is the concentrations of the gaseous reactants within the jar. These were determined through the gas exchange model (Equations (2)–(8)). The initial fractions of the anaerobic gas mixture were defined on the basis of the results obtained from the gas chromatography analysis, as follows: $\gamma_{Jar}=\left[0.21\;0.78\;0.01\;0.00\;0.00\right],\;\gamma_{Cylinder}=\left[0.00\;0.84404\;0.00\;0.04916\;0.1068\right]$, $M_{Gases}=\left[31.998\;28.014\;39.948\;2.015\;44.01\right]$, $V_{Jar}=0.00238483\;m^{3}$, R=8.314, T=273.15 K, predefined vacuum value $P_{Vac}=80,000\;Pa$, and $P_{Atm}=101,325\;Pa$. Figure 9 illustrates the graphical presentation of the calculated concentration changes of the jar as a function of the cycles.

As illustrated in the provided figure, the oxygen level in the jar atmosphere reaches 4.953 percent at the end of the first evacuation and replacement cycle, which is suitable for microaerophilic cultivation. Following the third cycle, the remaining oxygen level in the jar is approximately 0.2 percent, indicating an anaerobic condition. These findings are consistent with the methodology proposed by McIntosh and Fildes. Furthermore, the ABACUS control unit managed the cycles based on the same mathematical statements during the clinical validation tests. In light of the successful cultivation results outlined in Section 3.1.2, the calculations can be regarded as reliable.

**Figure 10:**
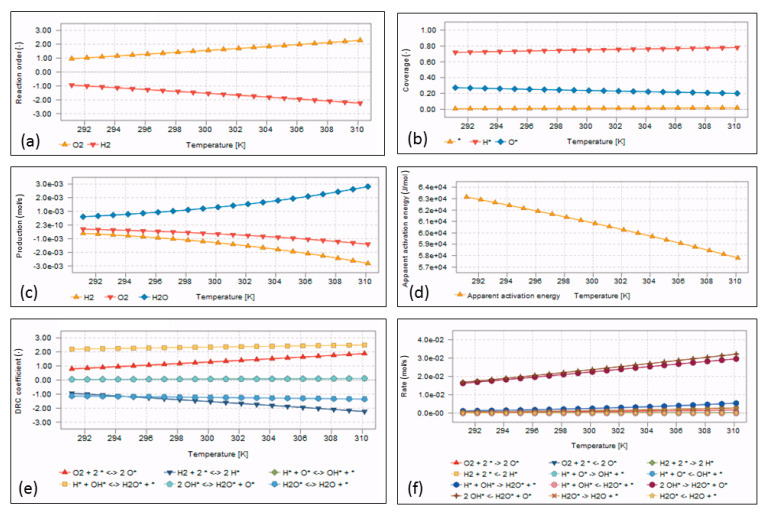
Microkinetic model results: (**a**) Reaction orders; (**b**) Surface coverage graphs; (**c**) Production graphs; (**d**) Apparent activation energy plot; (**e**) DRC coefficients; (**f**) Rates of elementary reactions.

In accordance with the conventional jar gassing systems, activity of palladium catalysts was observed when the catalyst test step was in progress. Turning over and microkinetic model calculations were conducted via the gaseous atmosphere formed at this stage. The catalyst test step is usually performed during the first replacement phase and the jar is filled to approximately 0.8 percent of atmospheric pressure. In our study, this replacement vacuum threshold was specified as 200 ± 10 mbar. In order to calculate concentrations, a vacuum pressure value was obtained from a real-time system (20,700 Pa). The normalized partial pressures of gaseous compounds used in the model are given as 0.047, 0.834, 0.00223, 0.0388, 0.0776 for O_2_, N_2_, Ar, H_2_, and CO_2_, respectively. The results of the microkinetic model simulation, generated using the given values, are depicted in Figure 10.

Microkinetic model graphics were plotted with the *x*-axis as temperature. Contrary to common practice in heterogeneous catalysis studies, temperature values have been scaled within a limited range covering room and incubation temperatures. This is an application-specific preference. One of the major findings that can be stated from microkinetic model outputs is the rate-determining step. The RDS is an essential component of Langmuir–Hinshelwood-type surface reaction mechanisms. The MKMCCX software(version 2.15.3) bundle implements the degree of rate control (DRC) methods that were introduced from Campbell [77] to identify the RDS step. The calculated DRC coefficients can be seen in Figure 10e. In contrast to recombination reactions generated on the Pt surface [47,78] at temperatures above 500 K, especially at lower temperature ranges, as in our case, the $H^{*}+{OH}^{*}⇄\;H_{2}O^{*}$ reaction has been reported as the RDS step [46,79] for surface reactions that occur on Pd(111) facets. The $O_{2}+2^{*}⇄2\;O^{*}$ is another key reaction that can be observed with the RDS step. Our findings are consistent with the values provided in the existing literature.

Figure 10c clearly demonstrates that the consumption rates of O_2_ and H_2_ are stoichiometrically meaningful. When analyzing reactions that occur over a wide temperature range, the particular temperature at which the apparent activation energy is zero can be considered the optimal level of the overall reaction rate. Nevertheless, as illustrated in Figure 10d, the apparent activation energy is positive and showed a slight variation of approximately 5000 J mol^−1^ across the specified temperature range of the microkinetic model. From Figure 10b, the surface is mostly blocked by H* species. In addition, the coverage graph indicates that the generation of vacant sites is quite limited due to the low temperature range [80,81]. This coverage pattern was frequently observed in the clinical validation tests, especially during the repeated jar gassing procedures due to the malfunctions of system components (e.g., jar leakage test error). In such cases, the catalytic activity of palladium pellets is significantly diminished unless they are exposed to atmospheric air for an adequate period between jar gassing procedures. Thus, the sachets were left exposed to the atmosphere for five minutes prior to reuse.

### 4.4. Analysis of the Extremum Seeking Algorithm

The convergence characteristics of the discrete extremum seeking algorithm used to establish a link between the real-time behavior of our catalytic system (ABACUS) and DFT-based microkinetics are elucidated in this section.

As frequently experienced in modeling studies, the model outputs cannot completely represent the actual response of the modeled system. In our case, there are several aspects that need to be considered about this issue. The majority of microkinetic model parameters were calculated using DFT findings, which offer very close values to operating surface analysis results, but these calculations are performed under high-vacuum conditions with a very limited molecule structure, which leads to inaccuracies. Furthermore, kinetic simulations were run with zero coverage postulated first, but due to our low temperature ranges, the collusion activity of gaseous reactants decreases, and these circumstances cause poisoning on the actual catalyst surface. As a consequence, reaction rates are reduced. To evaluate these aspects and emphasize the rate differences, a series of measurements were conducted by the ABACUS system during the clinical applications. The results of these tests are summarized in Table 8.

The measurements were taken different times but performed in the same laboratory environment, and environmental temperature fluctuations were measured between 22 °C and 23 °C. The minimum and maximum catalyst test intervals were utilized. Before testing, all sachets were baked at 180 °C to discharge. The fresh catalysts were used in the iterative jar gassing process. Based on the TOF values presented in Table 8, the following conclusions were reached. Despite the uniformity of the pellet volume within each sachet, the initial usage of the sachets indicated varying reaction rates, which were attributed to heterogeneities in the porous surface and transition metal doping ratio. Since there is no constant gas flow in the jar, the number of Petri dishes within the jar and their positions affect the natural convection of anaerobic gas in the jar atmosphere. This circumstance also manipulated the catalytic performance of the sachets. Furthermore, the poisoning effect can be seen from the results of Tests 4 through 6.

The working principle of the optimization algorithm was also visualized via these measurements. The extremum seeking coefficients used to identify the Arrhenius parameters of the example system (Test 4) selected from Table 8 are given as h=0.5, a=0.8, ξ=0.0135, $\alpha ={\left[20,\;12\times 10^{3}\right]}^{T}$, $\gamma ={\left[1000,\;15\times 10^{8}\right]}^{T}$. The TOF value of the associated test, recorded environment temperature, and reaction rate at 292 K, respectively, are as follows: $TOF=1.225\times 10^{-5}$, $T_{tof}=295.15\;K$, $k_{s@292K}=8.38\times 10^{-6}$. The initialization parameters of the extremum seeking algorithm determined via the microkinetic model are $\beta_{m}=1.03\times 10^{13}\;s^{-1}$, $E_{{act}_{m}}=95,535\;J\;{mol}^{-1}$(from RDS step). The *k_system_* was computed by taking the natural exponent of Equation (31).

**Figure 11:**
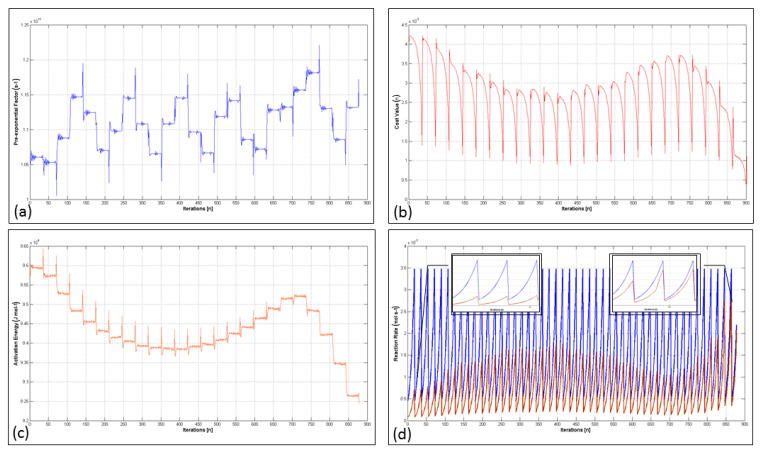
Extremum seeking convergence results: (**a**,**b**) Variations of Arrhenius parameters; (**c**) Cost function variation; (**d**) Convergence plot of the reaction rate.

The convergence results and tuning graphics of the optimization algorithm are presented in Figure 11. The algorithm was iterated based on the extremum seeking scheme outlined in Section 3.3 until the cost value reached the specified termination criteria. The parameters of the Arrhenius expression were updated at each iteration in accordance with the learning rate and cost value. The extremum seeking process was operated for each element of the *θ* vector, individually.

Figure 11d depicts the convergence between the measured and modeled reaction rate versus iterations. The variations observed in the *k_model_* can be seen over the subplots also shown in Figure 11d. Initial and final outputs are visualized in these plots. The extremum seeking algorithm was terminated after 878 iterations. At the last iteration, the Arrhenius parameters were recorded as $\theta ={\left[91,950,\;1.156\times 10^{13}\right]}^{T}$. Figure 11a,c illustrate the fluctuations of the pre-exponential factor and the activation energy, respectively. As is expected, the algorithm mostly modified the activation energy because it represents the exponential part of the reaction rate expression. The amplitude of the fluctuations is determined by the *γ* coefficient, and the selection of this parameter affects the seeking capability of the algorithm. In addition, the frequency of the perturbations affects the final iteration number and also defines the main trend of the seeker. The ripples in the needle shape observed in these plots were a consequence of the frequency of the perturbation signal and the discrete form of the algorithm. The variation in the cost function over iterations is shown in Figure 11b. Given the fluctuating but descending trend of the cost function output, one can easily state that the extremum seeking algorithm effectively minimizes the error gap.

## 5. Conclusions

The presented study provides comprehensive insights into anaerobic jar gassing systems, primarily from an engineering perspective. In order to achieve this objective, a fully functional control unit and essential system components were designed and manufactured. To ascertain the cultivation capabilities of the device, a series of atmosphere generation tests were conducted in parallel with a conventional anaerobic atmosphere creation pouch. Six distinct bacterial strains, encompassing both Gram-positive and Gram-negative anaerobic bacteria, were inoculated into agar plates. Furthermore, a *Campylobacter jejuni* strain was cultivated under microaerophilic conditions. The system was considered sufficient for basic cultivation purposes based on the proliferation outcomes. The anaerobic purification catalyst was examined in a computational chemistry and material science framework. During estimation of the microkinetic model parameters, the crystal structure of the Pd and Al_2_O_3_ was elucidated through XRD analysis, and the Pd(111), Pd(200), Pd(311), and Al_2_O_3_(220) refraction planes were identified. Additionally, the crystallite size of the planes was determined through the application of the Debye–Scherrer equation, and these findings were employed in the calculation of reaction rates. The chemical composition of the catalyst pellets was validated by EDS spectrogram. The eggshell structure was observed via cross-sectional SEM images, and the crust thickness of the Pd/Al_2_O_3_ catalyst was measured. The SEM results also revealed the presence of α-form Al_2_O_3_ crystallites, which correlated with the XRD peaks. Based on the morphological monitoring of the porous structure and the observation of needle-like XRD peaks, it was concluded that pellets constitute a catalytically active body, in accordance with the findings of studies on heterogeneous catalysis. The partial pressures of gases within the jar that vary based on the evacuation and replacement cycles were calculated via equations of the gas exchange model, and GC/MS results were used as input parameters. The learning-from-experiments approach that we employed to build our microkinetic model provided meaningful results when the real-time gassing tests were considered. To elevate the functionality of our jar gassing system, we have integrated a discrete extremum seeking algorithm into the device software, enabling the assessment of actual rate parameters for ongoing catalytic reactions. Considering the clinical importance of anaerobic cultivation systems, we conclude that such a comprehensive study on these systems will make a significant contribution to the literature. In addition, we believe that this research can serve as a roadmap for clinicians to identify the causes of technical failures during anaerobic cultivation.

## Figures and Tables

**Table 1:** A comparison of anaerobic cultivation methods.

Method ^(a)^	Material Consumption	Clinical Effectiveness	Application Area	Time Consumption for Preparation	Catalyst Requirement
Anaerobic Chamber	Reusable System	100% cultivation success ^(c)^	Large scale research or hospital laboratories	Less than 30 min (Ready to use after first run)	Required
Anaerobic Bag or Pouch	Disposable	80% cultivation success ^(b)^	Wide range applicability	Less than 5 min (Periodically)	Not required
Anaerobic Jar (Manual gas exchange)	Reusable System	90% cultivation success ^(b)^	Large and middle scale research or hospital laboratories	Less than 25 min (Periodically)	Required
Jar Gassing System (Automatic gas exchange)	Reusable System	100% cultivation success ^(b)^	Large and middle scale research or hospital laboratories	Less than 15 min (Periodically)	Required

^(a)^ [31]; ^(b)^ [32] Strictly anaerobic bacteria cultures were taken as reference; ^(c)^ Anaerobic chambers are standalone systems that provides continuous oxygen-free atmospheres.

**Table 2:** Catalyst specs.

Product Code	Pd Ratio [%]	Surface Area [m^2^ g^−1^]	Pore Volume [mL g^−1^]	Application Area
Deoxo D–4586	0.5	100 ^(a)^	0.35 ^(a)^	Removal of O_2_ from H_2_/N_2_ in anaerobic atmospheres

^(a)^ Approx.

**Table 3:** Results of GC/MS analysis.

Mixtures	Concentrations (%)			
	N_2_	O_2_	CO_2_	H_2_
Atmospheric Gas Mixture ^(a)^	81.051	18.949	0.000	0.000
Anaerobic Gas Mixture ^(b)^	84.404	0.000	10.680	4.916

^(a)^ Laboratory environment; ^(b)^ Connected to ABACUS system.

**Table 4:** Comparative bacterial cultivation results.

Bacterial Species	*ABACUS* ^(a)^		*GENBox* ^(b)^		Taxonomy ^(c)^
	Cell Viability	Colony Counts [CFU mL^−1^]	Cell Viability	Colony Counts [CFU mL^−1^]	
Bacteroides fragilis ^1^	High	1.34 × 10^5^	High	0.78 × 10^5^	Obligate Anaerob
Prevotella bivia ^2^	High	2.17 × 10^5^	High	2.95 × 10^5^	Obligate Anaerob
Cutibacterium acnes ^3^	High	1.77 × 10^5^	High	1.43 × 10^5^	Obligate Anaerob
Peptoniphilus asaccharolyticus ^4^	High	1.26 × 10^5^	High	1.18 × 10^5^	Obligate Anaerob
Finegoldia magna ^5^	High	2.05 × 10^5^	High	2.27 × 10^5^	Obligate Anaerob
Parvimonas micra ^6^	High	1.94 × 10^5^	High	2.35 × 10^5^	Obligate Anaerob

^(a)^ Our experimental system; ^(b)^ Single-use conventional system—bioMerieux^®^; ^(c)^ [31]; ^(1–6)^ Corresponds to the strain numbers in Figure 6.

**Table 5:** Calculated crystallite sizes.

2 Theta [°]	Reflected Planes [hkl]	FHWM [°]	Crystallite Size, L ^(a)^ [nm/Å]
38.3253	Pd(111)	0.24310	36.149/361.49
44.5257	Pd(200)	0.23150	38.476/384.76
64.8221	α-Al_2_O_3_(220)	0.25500	38.563/385.63
77.8653	Pd(311)	0.27160	39.296/392.96

^(a)^ Shape factor (*K*): 0.94.

**Table 6:** TST rate parameters.

Elementary Reaction Step	β_f_ [s^−1^]	E_f_ [J mol^−1^]	β_b_ [s^−1^]	E_b_ [J mol^−1^]
Equation (12)	1.228 × 10^13^	94,570	1.66 × 10^13^	97,369
Equation (13)	1.03 × 10^13^	47,285	1.03 × 10^13^	95,535
Equation (14)	4.97 × 10^13^	4825	5.166 × 10^13^	20,265
Equation (15)	7.288 × 10^15^	43,425	0.000	0.000

Note: 1 eV ~= 96.5 kJ mol^−1^; *f* and *b* are forward and backward, respectively.

**Table 7:** Hertz–Knudsen rate parameters.

Elementary Reaction Step	A_site_ [m^2^]	m [g]	θ_rot_^(a)^ [K]	σ ^(a)^ [-]	S [-]	E_des_ [J mol^−1^]
Equation (10)	4.37 × 10^−26^	32	2.1	2	1	77 × 10^3^
Equation (11)	4.37 × 10^−26^	2	87.9	2	0.5	96 × 10^3^

^(a)^ [76].

**Table 8:** Real-time measurements of reaction rates collected by ABACUS control unit.

Gassing Tests	TOF [mol s^−1^]	Δt_cat_test_ [s]	ΔP_loss_ [Pa]	T_tof_ [K]	Pd/Al_2_O_3_ Sachet Condition
Test 1, Sachet 1	1.633 × 10^−5^	90	1400	296.15	1st use
Test 2, Sachet 2	1.283 × 10^−5^	90	1100	295.15	1st use
Test 3, Sachet 3	1.633 × 10^−5^	90	1400	296.15	1st use
Test 4, Sachet 2	1.225 × 10^−5^	180	2100	295.15	2nd use
Test 5, Sachet 2	5.833 × 10^−6^	90	500	296.15	3rd use
Test 6, Sachet 2	9.917 × 10^−6^	180	1700	295.65	4th use

Note: The measured replacement vacuum threshold was observed to remain within the specified tolerance range. See Section 4.1.

## Data Availability

The data presented in this study are available within this article.
